# Epidemic Risk Perception, Perceived Stress, and Mental Health During COVID-19 Pandemic: A Moderated Mediating Model

**DOI:** 10.3389/fpsyg.2020.563741

**Published:** 2021-02-10

**Authors:** Xiaobao Li, Houchao Lyu

**Affiliations:** ^1^Faculty of Psychology, Southwest University, Chongqing, China; ^2^Time Psychology Research Center, Southwest University, Chongqing, China; ^3^China Community Psychology Service and Research Center, Southwest University, Chongqing, China

**Keywords:** future time perspective, confidence in society, coronavirus disease, epidemic risk perception, perceived stress, anxiety, depression

## Abstract

The aim of the present study was to investigate relationships among epidemic risk perception, perceived stress, mental health (depression and anxiety), future time perspective, and confidence in society during the novel coronavirus disease (COVID-19) pandemic in China. Especially, we wonder that whether perceived stress mediates associations between epidemic risk perception and mental health and that whether future time perspective and confidence in society moderate the link between perceived stress and mental health. This cross-sectional study was conducted among 693 Chinese adults aged 18–60 years. The results showed that epidemic risk perception was positively related to perceived stress, depression, and anxiety. The correlations between epidemic risk perception and depression and anxiety were reduced when perceived stress was included, suggesting that perceived stress mediated these relationships. Moreover, the boundary conditions for the associations among perceived stress, depression, and anxiety were found in the study. Specifically, positive future time perspective could buffer the negative effects of perceived stress on depression, and confidence in society could weaken the negative effects of perceived stress on anxiety. Based on these findings, practical guidance and theoretical implications are provided for the public to maintain mental health during COVID-19 pandemic. Limitations and future directions are also discussed.

## Introduction

The novel coronavirus disease (COVID-19) pandemic has spread across the globe. Owing to its rapid and extensive transmission, high infectivity, and lack of specific treatment so far, it has posed great threat to people’s mental and physical health. Unexpected public crisis events can easily cause the public to develop psychological reactions such as tension, anxiety, and even panic, which may lead to psychological disorders such as stress disorder and depression (Zhao et al., 2009). Therefore, it is of great practical significance and theoretical implications to study the impact of unexpected public crisis events on public mental health, and how to help individuals cope better with the crisis to maintain mental health.

Perceiving and avoiding risks are natural instincts of living beings. Risk perception is the core variable that induces psychological and behavioral responses among people in public crisis events, and exerts significant influences on both daily life decisions and behaviors (Slovic, 2000; Erdem and Swait, 2004; Li et al., 2009). Risk perception refers to an individual’s subjective judgment of risk based on objective crisis events, including the uncertainty about threats and severity of consequences (Cho and Lee, 2006). Perceived risk puts people in a distressed and anxious state, which in turn motivates them to engage in problem-solving activities to resolve it (Cho and Lee, 2006). People are likely to employ information search as a problem-solving strategy to reduce perceived risk, and they may also pay attention to existing problems and take precautions in advance to avoid more serious consequences (Shi et al., 2003; Li et al., 2009). However, if an individual stays in a highly threatening environment over a long period of time, certain physical and psychological problems tend to arise (Peters and McEwen, 2015; Nie et al., 2018). A study has revealed that people with a higher risk perception of the severe acute respiratory syndrome (SARS) epidemic are more likely to panic and respond unfavorably (Shi et al., 2003). A previous study on the Wenchuan Earthquake has shown that having a risk perception for unexpected natural disasters is negatively associated with public mental health (Li et al., 2009). Taking these together, we posit Hypothesis 1: Epidemic risk perception is positively related to depression (H1a) and anxiety (H1b).

The core of risk perception is the threat posed by uncertainties about the environment (Cho and Lee, 2006). In the context of an epidemic, people face huge uncertainties with respect to the life, work, economic prospects, and international relations. In such situations, the immediate feeling of the public is psychological stress. Perceived stress is the psychological response to threatening stimuli in the environment after cognitive evaluation and can be manifested as physical and mental tension, as well as loss of control (Cohen et al., 1983). The stress mainly stems from the sense of threat and expectation of adverse results in the future (Peters and McEwen, 2015; Peters et al., 2017). Individuals predict future results through comprehensive judgments of risk information in the present environment. If this prediction is filled with uncertainties, or if the expected results pose serious harm, stress will ensue, and even blood pressure may rise (Greco, 2003). Therefore, the higher the level of risk perception, the greater the psychological stress people will develop (Webster et al., 1988). This leads to the question that if risk perception causes stress, how does stress exert its impact on mental and physical health? Stress is one of the leading causes of mental and physical health problems (Gayman et al., 2011; Peters and McEwen, 2015). Research has shown that the body is likely to produce negative responses to cope with stress in a threatening environment (Gayman et al., 2011; Peters and McEwen, 2015), and increased stress is associated with many physical diseases (McEwen, 1998; Peters and McEwen, 2015) and mental health disorders, such as anxiety and depression (Olson and Surrette, 2004; Bardeen et al., 2013; Liu et al., 2015). Although researchers have investigated the relationship among risk perception, perceived stress, and mental health, few studies have been explored how risk perception affects mental health through the mediation of perceived stress. The risk perception of COVID-19 outbreak may lead to an increase in people’s perceived stress. On the one hand, people’s perception of uncertainty about the threat of being infected has caused them to maintain a stressful state. On the other hand, since the outbreak, a series of prevention and control measures such as lockdown of cities, road closures, work stoppages, and production shutdowns have exerted a huge impact on the daily life of the public. With the increased duration of preventive measures and lockdown, the circumstances of businesses being unable to resume work, sharp declines in individual incomes, fear of being infected, and inability to repay car loans and mortgages, etc., have imposed great psychological stress on people. In such a stressful environment, people become prone to allostatic overload, which could lead to negative psychological symptoms (Pedrelli et al., 2008). To sum up, we propose H2: Perceived stress mediates the link between epidemic risk perception and both depression (H2a) and anxiety (H2b).

The psychological resilience theory (Luthar et al., 2015) holds that individuals can successfully cope with stress and maintain mental health even in the face of adversity, because internal and external protective factors can alleviate the negative effects of stress on individuals. A research on which factors can help people cope with stress and respond with active adaptation for them to maintain mental health in times of crises carries great significance. Studies have pointed out that beliefs play an important role in human behaviors and mental health (Bandura, 1997; Luszczynska et al., 2009). People’s beliefs in the face of uncertainties moderate the intensity of stress responses (de Berker et al., 2016). The better one adjusts one’s beliefs in the face of uncertainties, the better the future results can be predicted, which in turn eases the stress response. With respect to protective factors within individuals, their beliefs and confidence in society constitute mental resources with which they respond effectively to environmental threats and uncertainties, and a high level of trust can reduce uncertainties and buffer the negative impact of environmental stress (Keller et al., 2011).

As a kind of important belief in the future, future time perspective (FTP) refers to an individual’s thought, feeling, and action tendencies toward the future (Lyu, 2014; Lyu and Huang, 2016). Individuals with a high level of FTP have three main characteristics: focusing on the future, being optimistic and cherishing hope, and valuing goals (Chin and Holden, 2013). Active attention to the future helps improve individuals’ life satisfaction (Pallini et al., 2016). When anticipating the future, an individual either looks forward to the future with optimism and bears confidence and hope for realizing future goals, or considers the future to be threatening (Ringle and Savickas, 1983). People with positive future orientation maintains an optimistic and hopeful attitude toward expectations of future results (Chin and Holden, 2013; Pallini et al., 2016), which can buffer the impact of environmental stress on negative emotions (Denovan and Macaskill, 2013) and reduce the tendency of depression (Hirsch et al., 2011). Individuals who value their future goals are able to predict the future values of their current behaviors, which stimulates their current adaptive behaviors (Chin and Holden, 2013). In the face of stressful situations, individuals with positive future orientation have strong adaptability, such as finding a job or shelter more quickly when they are homeless (Epel et al., 1999). Research on people’s mental health after the September 11 attacks have found that FTP is associated with higher levels of positive emotions (Holman, 2015; Holman et al., 2016) and reduced psychological stress 2 years post-9/11. In short, individuals with a positive attitude toward the future are able to maintain optimism and hope for the future, as well as focus on constructive behaviors that add to their future benefits. On the contrary, individuals with a negative attitude toward the future feel confused and pessimistic, which may easily lead to worry and anxiety about the future (Shipp et al., 2009). In summary, in this study, FTP is regarded as an important psychological resource that can buffer the adverse effects of perceived stress on mental health. Thus, H3 is proposed: Positive future time perspective negatively moderates the effect of perceived stress on both depression (H3a) and anxiety (H3b), while negative future time perspective positively moderates the effect of perceived stress on both depression (H3c) and anxiety (H3d).

Confidence in society refers to the positive expectation that society, based on its past performance, has its future under control (Keller et al., 2011). From a sociological perspective, confidence in society is a core component of social capital (Cook, 2005). If the members of a society have confidence in the ability of the social system to deal with future problems and risks, then social capital will increase. High levels of confidence in society make individuals feel calm and safe and show more cooperative behavior when responding to threats (Earle et al., 2007), so as to ensure the continuous operation of society (La Porta et al., 1997). From a psychological perspective, confidence in society is assumed to act as a psychological buffer against the influence of environmental stress and uncertainties evoked by societal transformation (Keller et al., 2011). Research has shown that confidence in society is negatively correlated with trait anxiety and neuroticism, and positively correlated with self-esteem, self-efficacy, and life satisfaction (Keller et al., 2011). Individuals lacking confidence in society do not believe that society can cope with crises and maintain control and stability, and they are likely to experience greater tension and anxiety. Thus, this study considers confidence in society as a psychological buffer against the effect of perceived stress and mental health. In a situation of lack of sufficient information to reduce fear of the unknown, high levels of confidence in society can effectively reduce anxiety and depression, thus buffering the negative effects of perceived stress on mental health. Although dealing with uncertainties brought by changes in the environment is essential to individuals’ life and mental health, little research has been conducted in this area so far. Therefore, we posit that H4: Confidence in society negatively moderates the effect of perceived stress on both depression (H4a) and anxiety (H4b).

In summary, the aim of the research is to investigated the mediating effect of perceived stress on the relationship between epidemic risk perception and mental health (anxiety and depression), and examine the moderating effect of FTP and confidence in society on the relationship between perceived stress and mental health. The validation of the protective effect of FTP and confidence in society may help to support the public in maintaining their mental health and carrying out psychological prevention and intervention during an epidemic from the perspective of positive psychology.

## Materials and Methods

### Participants and Procedure

During the outbreak of novel coronavirus disease (COVID-19) in China, we recruited participants via wjx.cn, a reliable Chinese online platform for data collection and randomly distributed questionnaire links in the participant pool. The data collection began on February 6, 2020. A week later, 701 participants answered the questionnaires. All participants consented to attend the study after being informed about the purpose of the study. After excluding cases with invalid responses (e.g., too-short answering time or same answers for each item), we retained a final sample of 693 participants. Participants were given a packet of questionnaires that included questions regarding demographics, epidemic risk perception, perceived stress, anxiety, depression, future time perspective, and confidence in society. No direct compensation was provided for study participation.

The samples of the present study were mainly from Henan Province (32.6%), Shandong Province (28.6%), and Chongqing city (29.9%), accounting for 91% of the total samples. Only 17 participants were from Wuhan, Hubei Province, and the remaining 45 participants were scattered in other Chinese cities. The sample consisted of 619 general public, 17 quarantined personnel, 29 frontline medical workers, and 12 community service workers. Among all participants, 62.0% were females and 38.0% were males. Moreover, 29.9% were between 18 and 25 years old, 18% were between 26 and 30 years old, 22.7% were between 31 and 40 years old, 21.2% were between 41 and 50 years old, and 8.2% were between 51 and 60 years old. Also, 82.4% of participants received at least a college degree.

### Measures

#### Epidemic Risk Perception

One single item was used to measure epidemic risk perception. Participants were asked to evaluate the perceived risk of infection during the outbreak of COVID-19. Ratings were given on a 10-point Likert scale (1 = not at all threatening, 10 = extremely threatening). In this study, the average score of epidemic risk perception of all participants was 6.03 (*SD* = 2.26).

#### Perceived Stress

The perceived stress scale-10 (PSS-10; Cohen et al., 1983) was used to measure the extent to which respondents feel that their stress is unpredictable, uncontrollable, and overwhelming. It comprises 10 items that allow five responses in a Likert scale: never (0), almost never (1), sometimes (2), often (3), and very often (4). Total scores range from 0 to 40, with higher scores indicating greater perceived stress. Cronbach’s alpha with the current sample was 0.85.

#### Mental Health

Anxiety and depression were used as indicators of mental health.

*Anxiety:* The generalized anxiety disorder-7 scale (GAD-7) was used to measure participants’ worry and general somatic tension (Spitzer et al., 2006). It has seven items rated on a four-point Likert scale indicating symptom frequency, ranging from 0 (not at all) to 3 (nearly every day). Higher scores indicate higher levels of anxiety symptoms. In this study, Cronbach’s alpha of the scale was 0.92.

*Depression:* The center for the epidemiological studies of depression-10 (CES-D-10; Andresen et al., 1994) was used to measure depression. This scale consists of 10 items to assess symptoms of depression (e.g., “I felt depressed”), and response anchors range from 0 (rarely) (less than 1 day) to 3 (most or all of the time) (5–7 days). Participants indicate how true each statement is to them over the past week. Cronbach’s alpha for the present sample was 0.84.

#### Future Time Perspective

Future time perspective was assessed by the future subscale of Time Attitude Scale (TAS, Worrell et al., 2013). The scale consists of 30 items on six subscales: past positive, past negative, present positive, present negative, future positive, and future negative, which has demonstrated adequate reliability, validity, and generally strong psychometric properties in adolescent and adult samples (Mello et al., 2016). In the present study, we mainly adopted the future dimension of TAS, with 10 items and 2 subscales (future positive and future negative). Participants were asked to answer the questions on a five-point scale from 1 (strongly disagree) to 5 (strongly agree). In the present sample, Cronbach’s alphas of future positive and future negative were 0.85 and 0.73, respectively.

#### Confidence in Society

Confidence in society was assessed by the general confidence scale developed by Keller et al. (2011). The scale has six items rated on a seven-point Likert scale (1 = totally disagree, 7 = totally agree). Higher scores indicate higher levels of confidence in society. In this study, Cronbach’s alpha score of the scale was 0.89.

### Control Variables

We controlled for participants’ gender (0 = female, 1 = male), age (1 = 18–25 years; 2 = 26–30 years; 3 = 31–40 years; 4 = 41–50 years; 5 = above 51 years), and education level (1 = vocational school, technical secondary school; 2 = high school; 3 = vocational/junior college; 4 = undergraduate; 5 = graduate) because these demographic variables have been reported to link to individuals’ mental health (e.g., Cauce et al., 2000; Halpern-Manners et al., 2016).

### Data Analysis

Statistical analysis was performed using SPSS 22.0 and AMOS 21.0 software packages. The normal distribution of all variables was tested using the Kolmogorov–Smirnov test, and all continuous variables follow the normal distribution. The statistical methods included descriptive statistics, correlation analysis, regression analysis, structural equation model, and bootstrap analysis, etc. The significance level of all variables was set as α = 0.05.

## Results

### Descriptive Statistics and Correlation Analyses

Descriptive statistics, including means, SDs, correlations, and reliabilities, are presented in Table 1. Epidemic risk perception, perceived stress, anxiety, and depression were found to be positively related to one another. Moreover, future negative was positively associated with anxiety and depression. Future positive and confidence in society were negatively linked with anxiety and depression.

**TABLE 1:** Means, SDs, and correlations.

Variables	1	2	3	4	5	6	7	8	9	10
1. Epidemic risk perception	–									
2. Perceived stress	0.19**	*0.85*								
3. Future positive	−0.07	−0.54**	*0.85*							
4. Future negative	0.10*	0.48**	−0.58**	*0.74*						
5. Confidence in society	−0.07	−0.41**	0.53**	−0.34**	*0.89*					
6. Anxiety	0.29**	0.63**	−0.30**	0.30**	−0.26**	*0.92*				
7. Depression	0.17**	0.73**	−0.53**	0.48**	−0.40**	0.71**	*0.84*			
8. Gender	0.06	0.08*	−0.01	0.04	0.03	0.06	0.03	–		
9. Age	0.11**	−0.23**	0.12**	−0.01	0.15**	−0.02	−0.16**	−0.25**	–	
10. Education level	−0.04	−0.04	0.03	−0.10**	−0.01	−0.08*	−0.06	0.03	−0.05	–
*M*	6.03	2.68	3.69	2.33	5.32	1.29	1.82	0.38	3.59	4.04
*SD*	2.26	0.68	0.78	0.83	1.19	0.42	0.48	0.49	1.36	0.9

N = 693. Cronbach’s alphas are presented on the diagonal in italics. Gender: 0, female; 1, male. Education: 1, vocational school, technical secondary school; 2, high school; 3, vocational/junior college; 4, undergraduate; 5, graduate. Age: 1, 18–25 years; 2, 26–30 years; 3, 31–40 years; 4, 41–50 years; 5, above 51 years. *p < 0.05, **p < 0.01.

### Associations Between Epidemic Risk Perception and Mental Health

In the current study, regression analysis was used to explore the association between epidemic risk perception and mental health. Hypotheses 1a and 1b posit that epidemic risk perception is positively related to depression (H1a) and anxiety (H1b). As shown in Table 2, after the effects of gender, age, and education level had been controlled, epidemic risk perception positively related to depression (β = 0.19, *SE* = 0.04, *p* < 0.01) and anxiety (β = 0.28, *SE* = 0.04, *p* < 0.01). Thus, Hypothesis 1a and 1b were supported.

**TABLE 2:** Results of regression analysis.

	Depression	Anxiety
**Control variables**		
Gender	−0.03 (0.04)	0.031 (0.04)
Age	−0.18 (0.04)**	−0.050 (0.04)
Education level	−0.06 (0.04)	−0.074 (0.04)*
**Predictor**		
Epidemic risk perception	0.19 (0.04)**	0.28(0.04)**
*F*	11.95**	17.49**
Adjusted *R*^2^	0.07	0.09

N = 693. Statistics reported are standardized regression coefficients (and SEs). *p < 0.05, **p < 0.01.

### Examination of Moderated Mediation Model

More also, path analysis was conducted in Amos21.0 to test the mediating effect of perceived stress between epidemic risk perception and mental health, and the moderating effect of FTP and confidence in society on the relationship between perceived stress and mental health. Given that anxiety and depression were used as indicators of mental health, we developed two models with anxiety (Model 1) and depression (Model 2) as outcome variables, respectively. Both model 1 and model 2 had a reasonably good fit to the data [Model 1: χ^2^/*df* = 2.30, comparative fit index (CFI) = 0.98, Tucker–Lewis index (TLI) = 0.96, root mean square residual (RMR) = 0.04, root mean square error of approximation (RMSEA) = 0.04; Model 2: χ^2^/*df* = 2.29, CFI = 0.98, TLI = 0.96, RMR = 0.04, RMSEA = 0.04]. Table 3 shows the results of path analysis of the hypothesized model.

**TABLE 3:** Path analysis results on depression and anxiety.

	Model 1		Model 2	
	Perceived stress	Depression	Perceived stress	Anxiety
		
	β (*SE*)	β (*SE*)	β (*SE*)	β (*SE*)
**Control variables**				
Gender	0.02 (0.03)	−0.04 (0.02)	0.02 (0.03)	0.03 (0.03)
Age	−0.18** (0.03)	−0.02 (0.03)	−0.18** (0.03)	0.10** (0.03)
Education level	−0.01 (0.03)	−0.02 (0.02)	−0.01 (0.03)	−0.05 (0.03)
**Predictors**				
Epidemic risk perception	0.16** (0.03)	0.04 (0.03)	0.16** (0.03)	0.15** (0.03)
Perceived stress		0.62** (0.03)		0.67** (0.04)
Future positive		−0.09*(0.03)		−0.07 (0.04)
Future negative		0.10** (0.03)		0.03 (0.04)
Confidence in society		−0.07* (0.03)		−0.02 (0.03)
Future positive × perceived stress		−0.15** (0.03)		−0.06 (0.04)
Future negative × perceived stress		−0.02 (0.03)		−0.04 (0.04)
Confidence in society × perceived stress		−0.01 (0.03)		−0.11** (0.03)

N = 693. Statistics reported are standardized regression coefficients (and SEs). *p < 0.05, **p < 0.01.

Hypotheses 2a and 2b predict that the positive relationships between epidemic risk perception and depression/anxiety are mediated by perceived stress. As shown in Table 3, epidemic risk perception was found to be positively related to perceived stress (β_*Model* 1_ = 0.16, *SE* = 0.03, *p* < 0.001; β_*Model* 2_ = 0.16, *SE* = 0.03, *p* < 0.001), and perceived stress was positively related to depression (β = 0.62, *SE* = 0.03, *p* < 0.001, Model 1) and anxiety (β = 0.67, *SE* = 0.04, *p* < 0.001, Model 2). When perceived stress was included, epidemic risk perception positively related to anxiety (β = 0.15, *SE* = 0.03, *p* < 0.001, Model 2) but not related to depression (β = 0.04, *SE* = 0.03, *p* < 0.05, Model 1). These results suggest that perceived stress partially mediates the link between epidemic risk perception and anxiety, and fully mediates the link between epidemic risk perception and depression. We further tested the two mediating effects using 5,000 bootstrapping samples. The analyses indicated a significant mediating effect between epidemic risk perception and depression through perceived stress [indirect effect = 0.10, *SE* = 0.02, 95% CI (0.06, 0.14), excluding zero]. The results also indicated a significant mediating effect between epidemic risk perception and anxiety through perceived stress [indirect effect = 0.10, *SE* = 0.02, 95% CI (0.07, 0.15), excluding zero]. Thus, Hypotheses 2a and 2b were supported.

**FIGURE 1:**
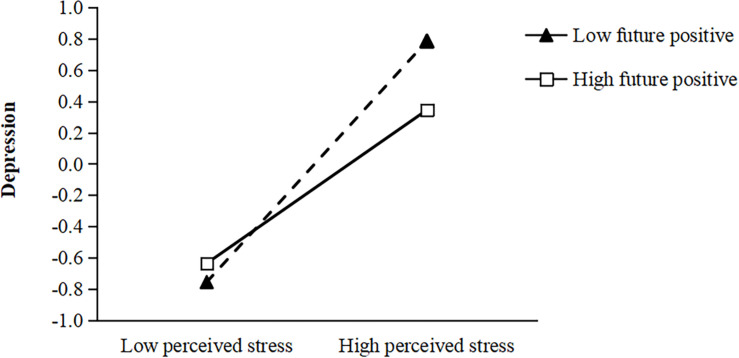
Interactive effects of future positive and perceived stress on depression.

Hypotheses 3a, 3b, 3c, and 3d propose the moderating effect of future positive/future negative on the relationship between perceived stress and depression/anxiety such that the relationships become weaker when future positive is high rather than low and when future negative is low rather than high. We centered all continuous variables before creating their product terms. The results from path analysis show that only the interaction term of future positive and perceived stress is negatively related to depression (β = 0.15, *SE* = 0.03, *p* < 0.001; Model 1). To further interpret the results, we conducted a simple slopes analysis. The interaction plot in Figure 1 shows that with low future positive (1 SD below the mean), perceived stress is negatively related to depression (*simple slope* = 0.79, *SE* = 0.04, *p* < 0.001) and stronger, while with high future positive (1 SD above the mean), perceived stress is significantly related to depression (*simple slope* = 0.55, *SE* = 0.03, *p* < 0.001) and weaker. Thus, Hypothesis 3a was supported, but hypotheses 3b, 3c, and 3d were not supported.

**FIGURE 2:**
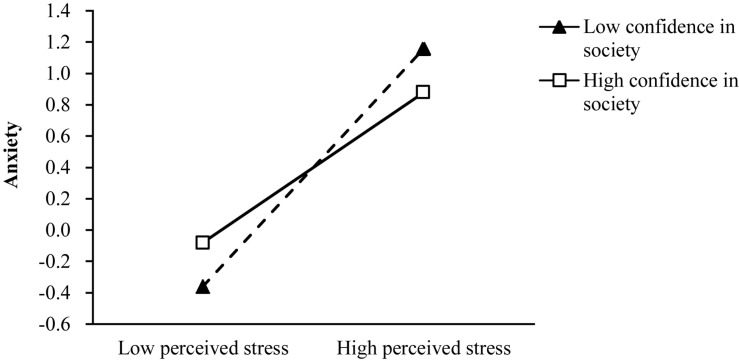
Interactive effects of confidence in society and perceived stress on anxiety.

Hypotheses 4a and 4b predict that the positive relationships between perceived stress and depression/anxiety are negatively moderated by confidence in society such that the relationships become weaker when confidence in society is high rather than low. The results from path analysis indicated that only the interaction term of confidence in society and perceived stress was negatively related to anxiety (β = 0.11, *SE* = 0.03, *p* < 0.05; Model 2). The interaction plot in Figure 2 indicates that with low confidence in society (1 SD below the mean), perceived stress was negatively related to anxiety (*simple slope* = 0.79, *SE* = 0.05, *p* < 0.001) and stronger, while with high confidence in society (1 SD above the mean), perceived stress was significantly related to anxiety (*simple slope* = 0.51, *SE* = 0.04, *p* < 0.001) and weaker. Thus, hypothesis 4a was not supported and hypothesis 4b was supported.

## Discussion

This study investigated the mediating effect of perceived stress on the relationship between epidemic risk perception and mental health (anxiety and depression), and the moderating effects of FTP and confidence. The results revealed that epidemic risk perception has a significant positive effect on anxiety and depression, which is consistent with previous research results on public crisis events and mental health (Shi et al., 2003; Li et al., 2009; Nie et al., 2018). The occurrence of public crisis events sharply increases individuals’ perceived risks, and such an environment, filled with threats and uncertainties, can easily cause anxiety and depression (Zhao et al., 2009; Peters and McEwen, 2015). The risks during an epidemic also lead to a loss of the sense of control and a feeling of powerlessness, wherein the public can only passively await the development of the epidemic, and people may thus experience higher level of depression and anxiety.

More importantly, this study found that perceived stress exerts a partial mediating effect on the relationship between epidemic risk perception and anxiety, and a full mediating effect on the relationship between epidemic risk perception and depression. This difference may be caused by the different cognitive features of anxiety and depression. Given that the cognitive bias of depression is a combination of emotions and negative memories, people with excessive psychological stress tend to indulge in the negative emotions and unable to escape; therefore, perceived stress may fully mediate the association between epidemic risk perception and depression. However, the cognitive features of anxiety is characterized by excessive attention bias to specific negative stimuli, reflecting the activity of the fear system, thus there may have been other factors such as worries about the future that also could explain the link between epidemic risk perception and anxiety. The results of the present study demonstrate perceived stress is the main underlying mechanism that explains the effect of perceived risk on mental health. When people are exposed to negative life events, such as the COVID-19 pandemic, concerns about current terrible situations and future adverse consequences may lead to a lot of psychological stress, which in turn activate an individual’s diathesis or vulnerability, transforming potential diathesis into a reality of psychopathology (Monroe and Simons, 1991). The relationships among stress, anxiety, and depression mainly lie in the subjective perception of pain and lack of ability to cope with stress (Hewitt et al., 1992). If stress could be effectively alleviated, negative emotions and mental symptoms will be avoided easily.

The current study found that FTP moderates the relationship between perceived stress and depression. Compared with individuals with negative future orientation, those with positive future orientation are relatively weaker in perceiving the impact of stress on depression. According to the diathesis-stress theory (Monroe and Simons, 1991), perception of future self and the world is the direct cause of depression. Individuals with a position future orientation can maintain optimism and hope for the future when an epidemic occurs (Chin and Holden, 2013; Pallini et al., 2016). Thus, they are likely to have more adaptable behaviors in stressful situations, which contribute to alleviate the effects of stress on depression. However, individuals with a negative future orientation hold a negative attitude toward the future. When facing stressful situations, they will be easily trapped in the pain of the past and present, further exacerbating their depression (Holman et al., 2016).

In this study, we found that confidence in society can moderate the relationship between perceived stress and anxiety. Individuals with high confidence in society can avoid excessive anxiety under environmental threats and stress. According to Bandura (1997) and Luszczynska et al. (2009), beliefs (such as self-efficacy) play an important role in human behaviors and mental health, and confidence in society is similar to a sense of collective efficacy, which is an individual’s positive belief in society’s ability to deal with threats (Keller et al., 2011). The unexpected COVID-19 pandemic brought varied risks and dangers in people’s lives, and has become a source of uncertainties and tension. In such a setting, individuals with high confidence in society have positive expectations for the future, and believe that the society is capable of coping with threats to achieve a sense of certainty and control, thereby avoiding tensions and maintaining calm. In contrast, individuals with low confidence in society are more emotionally vulnerable, and tend to be nervous and anxious when faced with an epidemic. It should be noted that confidence in society is not an irrational belief but a positive illusion that makes it easier for people to cope with difficult life situations (Bandura, 1998). Individuals with high confidence in society can fully recognize the causes of danger without overestimating risks, and thus are able to remain calm and are more likely to take effective preventive measures as necessary.

In sum, the present study supports that perceived stress mediates the link between epidemic risk perception and mental health, and that FTP and confidence in society are both important variables of psychological buffer with which individuals deal with stress from the epidemic and effectively reduce the adverse effects of perceived stress on mental health. These results help to better explain how and when epidemic risk perception leads to depression and anxiety and provide theoretical guidance and inspiration for studies on epidemic intervention. First, perceived stress is an important mechanism by which the risk perception of unexpected public crisis event affects mental health, and stress relief is an important means for reducing mental problems. It is well known that fear stems from uncertainties; therefore, helping people gain necessary epidemic knowledge about COVID-19, such as epidemic characteristics, prevention and control measures, etc., would transform uncertainties into understanding, thereby correcting the perception of threat events and false beliefs to better predict future results and cope with environmental threats (Peters et al., 2017). Previous studies have found that social skills are correlated with a decrease in stressful experiences, and that people with strong social skills gain more social supports when faced with stress (Segrin et al., 2007); therefore, they should communicate with family and friends over the phone or the Internet to encourage one another and strengthen mutual mental support to alleviate tension and psychological stress. Second, correct understanding of the impact of the epidemic and a positive belief in the future are effective ways to reduce depression. Crises are always accompanied by dangers as well as opportunities. Although the COVID-19 pandemic has caused huge losses, it has also reminded all sectors of society to pay attention to physical and mental health and the prevention and control of epidemics, which offers experiences of reference value for similar events in the future. Individuals should strengthen self-management and adjustment, adopt a dialectical approach to crises, and establish a correct and positive conception of the future to maintain mental health. Furthermore, it is necessary to attach importance to the improvement and cultivation of confidence in society. The public’s judgment on confidence in society is mainly based on the past performance of the society (Keller et al., 2011) as well as the positive role of the government and the media. When individuals feel a lack of control during an epidemic, the government and social organizations should provide sufficient guarantees and supports to allow them to feel that society is still functioning with order and certainty, thereby avoiding anxiety and panic. In addition, the media’s coverage of crisis events is the main source from which people obtain epidemic-related information. Negative news reports often lead to negative emotional experiences among people. Therefore, the media should pay attention to positive and favorable news about responses to the crisis from all walks of life, and ensure objectivity and scientific nature of media information in guiding people to correctly understand the impact of epidemic crisis events, and cultivate optimism, positive emotions, and positive attitudes toward the future.

Despite its strengths, this study has some limitations. With a questionnaire survey targeting the nationwide public, only about 700 entries of data were collected in the study. As the sample size was relatively small, the distribution was uneven with respect to region (small sample from Hubei province), education level (bachelor’s degree and above accounted for 82.3%), and personal status in the epidemic (few participants representing those in quarantine, frontline medical workers, and community service workers). Second, the study was not conducted in special regions and among special groups. Future research should pay more attention to people in quarantine, frontline medical workers, community service personnel, etc. For instance, more attention should be paid to the mental health of people in Wuhan, as the COVID-19 started in Wuhan and most of the infected cases in China were also found in Wuhan. Instead of self-isolation of other areas, people in Wuhan were forcibly quarantined to confirm whether they have become sick and minimize the risk of them passing on the infection to others. During the period of the quarantine, fear of infection with a fatal disease, the lack of information, frustration and boredom, lack of supplies, and not being able to go to work or earn an income could lead to problems in both mental and physical health (Brooks et al., 2020). People in Wuhan also suffer a lot of social stigma, which may lead to worse psychological problems than people in other regions. Third, only one item was used to measure overall risk perception in the present study. While it could be useful to use an item to measure overall risk perception, the current study did not distinguish the effects of different aspects of risk perception (uncertainty about threats and severity of consequences) on perceived stress and mental health. Future research could benefit from improving this tool. Finally, as cross-sectional design was used, this study did not track the mental health of people during the epidemic and had inadequate understanding of the public’s emotional or mental changes during the period of epidemic outbreak. Therefore, causal relations among variables could not be confirmed, and future research should adopt longitudinal design and interventional experiments to provide better assistance for building mental health in times of crises.

## Conclusion

The present study demonstrated that risk perception of COVID-19 was significantly correlated with depression and anxiety. Perceived stress was established as a mediator of epidemic risk perception and depression/anxiety. Future time perspective was found to moderate the effect of perceived stress on depression and social confidence was found to moderate the effect of perceived stress on anxiety.

## Data Availability Statement

The datasets generated for this study are available on request to the corresponding author.

## Ethics Statement

The studies involving human participants were reviewed and approved by the Ethics Committee of Faculty of Psychology at Southwest University. The patients/participants provided their written informed consent to participate in this study.

## Author Contributions

HL and XL designed the study idea and research framework. XL contributed to the data collection, data analysis, and writing. HL contributed to manuscript writing and modification. Both authors read and approved the manuscript.

## Conflict of Interest

The authors declare that the research was conducted in the absence of any commercial or financial relationships that could be construed as a potential conflict of interest.
